# Bioinformatic analysis suggests that the Orbivirus VP6 cistron encodes an overlapping gene

**DOI:** 10.1186/1743-422X-5-48

**Published:** 2008-04-14

**Authors:** Andrew E Firth

**Affiliations:** 1 Department of Biochemistry, BioSciences Institute, University College Cork, Cork, Ireland

## Abstract

**Background:**

The genus *Orbivirus *includes several species that infect livestock – including Bluetongue virus (BTV) and African horse sickness virus (AHSV). These viruses have linear dsRNA genomes divided into ten segments, all of which have previously been assumed to be monocistronic.

**Results:**

Bioinformatic evidence is presented for a short overlapping coding sequence (CDS) in the *Orbivirus *genome segment 9, overlapping the VP6 cistron in the +1 reading frame. In BTV, a 77–79 codon AUG-initiated open reading frame (hereafter ORFX) is present in all 48 segment 9 sequences analysed. The pattern of base variations across the 48-sequence alignment indicates that ORFX is subject to functional constraints at the amino acid level (even when the constraints due to coding in the overlapping VP6 reading frame are taken into account; MLOGD software). In fact the translated ORFX shows greater amino acid conservation than the overlapping region of VP6. The ORFX AUG codon has a strong Kozak context in all 48 sequences. Each has only one or two upstream AUG codons, always in the VP6 reading frame, and (with a single exception) always with weak or medium Kozak context. Thus, in BTV, ORFX may be translated via leaky scanning. A long (83–169 codon) ORF is present in a corresponding location and reading frame in all other *Orbivirus *species analysed except Saint Croix River virus (SCRV; the most divergent). Again, the pattern of base variations across sequence alignments indicates multiple coding in the VP6 and ORFX reading frames.

**Conclusion:**

At ~9.5 kDa, the putative ORFX product in BTV is too small to appear on most published protein gels. Nonetheless, a review of past literature reveals a number of possible detections. We hope that presentation of this bioinformatic analysis will stimulate an attempt to experimentally verify the expression and functional role of ORFX, and hence lead to a greater understanding of the molecular biology of these important pathogens.

## Background

The *Orbivirus *genus is one of ≥12 genera within the family *Reoviridae*. The *Reoviridae *have segmented linear dsRNA genomes. There are 9–12 segments [1] and these are usually, but not always, monocistronic. Subgenomic RNAs are unknown. *Orbivirus *genomes have 10 segments. Many species infect ruminants while some infect humans. Transmission is via arthropods – including midges, ticks and mosquitoes. The type species is Bluetongue virus (BTV) which causes severe and sometimes fatal disease, particularly in sheep. BTV is endemic in many tropical countries, but there have also been recent outbreaks in Europe [2,3]. Another species is African horse sickness virus (AHSV) which is a fatal disease of horses. AHSV is endemic in many parts of sub-Saharan Africa, but has made incursions into Europe [4]. Recent outbreaks of BTV in Europe may be a consequence of climate change – allowing the midge vectors to expand their range [5].

The *Orbivirus *proteins, structure, assembly and replication have been reviewed in [6-8]. The BTV core is composed of two major proteins (VP3 and VP7). Transcription complexes – composed of three minor proteins (VP1 – polymerase, VP4 – capping enzyme, and VP6 – helicase) are located inside the core. Transcription occurs within the intact core and full-length capped mRNAs from each of the genome segments are fed out into the cytoplasm for translation. An outer capsid (VP2 and VP5) surrounds the core, but is removed during cell entry. There are four non-structural proteins – NS1, NS2 and NS3/3A. VP6 is a hydrophilic, basic protein that binds dsRNA and other nucleic acids and functions as the viral helicase [9-13]. In some, but not all, BTV serotypes, VP6 migrates as a closely-spaced doublet [14]. This is apparently due to the fact that in these serotypes the first VP6 AUG codon has weak Kozak context while a second in-frame AUG codon has medium context.

The genomes of RNA viruses are under strong selective pressure to compress maximal coding and regulatory information into minimal sequence space. Thus overlapping CDSs are particularly common in such viruses. Such CDSs can be difficult to detect using conventional gene-finding software [15], especially when short. The software package MLOGD, however, was designed specifically for locating short overlapping CDSs in sequence alignments and overcomes many of the difficulties with alternative methods [15,16]. MLOGD includes explicit models for sequence evolution in double-coding regions as well as models for single-coding and non-coding regions. It can be used to predict whether query ORFs are likely to be coding, via a likelihood ratio test, where the null model comprises any known CDSs and the alternative model comprises the known CDSs plus the query ORF. MLOGD has been tested extensively using thousands of known virus CDSs as a test set, and it has been shown that, for overlapping CDSs, a total of just 20 independent base variations are sufficient to detect a new CDS with ~90% confidence.

Using MLOGD, we recently identified – and subsequently experimentally verified – a new short CDS in the *Potyviridae *that overlaps the polyprotein cistron but is translated in the +2 reading frame [17]. When we applied MLOGD to the *Orbivirus *genome we also found evidence for a short CDS overlapping the VP6 cistron. Here we describe the bioinformatic analysis.

## Results

### Identification in BTV using MLOGD

**Figure 1:**
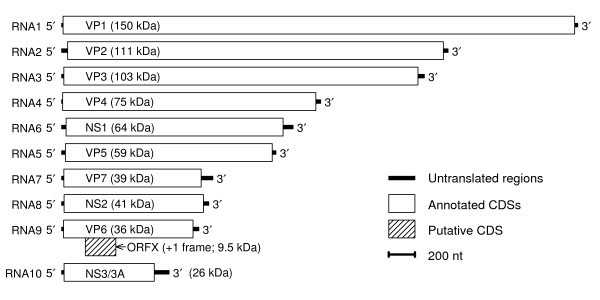
**Genome map for BTV**. The putative new coding sequence – ORFX – is located on segment 9 (RNA9), in the +1 reading frame relative to the overlapping VP6 cistron. Molecular masses are based on the unmodified amino acid sequences.

**Figure 2:**
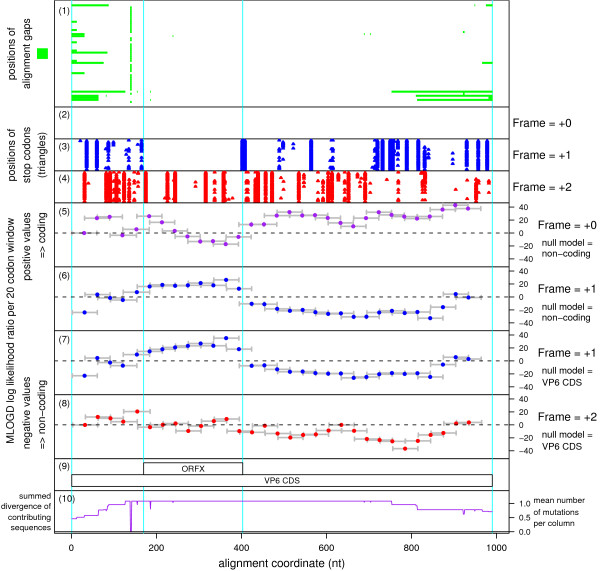
**MLOGD statistics for the alignment of 48 BTV sequences**. The input alignment comprised a CLUSTALW [39] alignment of the VP6 amino acid sequences only, back-translated to nucleotide sequences. **(1) **The positions of alignment gaps in each of the 48 sequences. In fact most of the alignment is ungapped, though a few sequences are incomplete. **(2)–(4) **The positions of stop codons in each of the 48 sequences in each of the three forward reading frames. Note the conserved absence of stop codons in the +0 frame (i.e. the VP6 CDS) and in the +1 frame in the ORFX region. **(5)–(8) **MLOGD sliding-window plots. Window size = 20 codons. Step size = 10 codons. Each window is represented by a small circle (showing the likelihood ratio score for that window), and grey bars showing the width (ends) of the window. See [16] for further details of the MLOGD software. In **(5)–(6) **the null model, in each window, is that the sequence is non-coding, while the alternative model is that the sequence is coding in the window frame. Positive scores favour the alternative model. There is a strong coding signature in the +0 frame (5) throughout the VP6 CDS, except where the VP6 CDS overlaps ORFX. In this region there is a strong coding signature in the +1 frame (6) indicating that ORFX is subject to stronger functional constraints than the overlapping section of VP6. In **(7)–(8) **the null model, in each window, is that only the VP6 frame is coding, while the alternative model is that both the VP6 frame and the window frame are coding. Only the +1 (7) and +2 (8) frames are shown because the +0 frame is the VP6 frame which is included in the null model. Scores are generally negative with occasional random scatter into low positive scores, except for the ORFX region which has consecutive high-positively scoring windows (7). Note that there are four non-overlapping – and hence completely independent – positively scoring windows in the ORFX region (7). Formally, and within the MLOGD model, *p *< 10^-40^. **(9) **Genome map for the reference sequence [GenBank: NC_006008]. **(10) **Phylogenetically summed sequence divergence (mean number of base variations per nucleotide) for the sequences that contribute to the statistics at each position in the alignment. In any particular column, some sequences may be omitted from the statistical calculations due to alignment gaps. Statistics in regions with lower summed divergence (i.e. partially gapped regions) have a lower signal-to-noise ratio.

**Figure 3:**
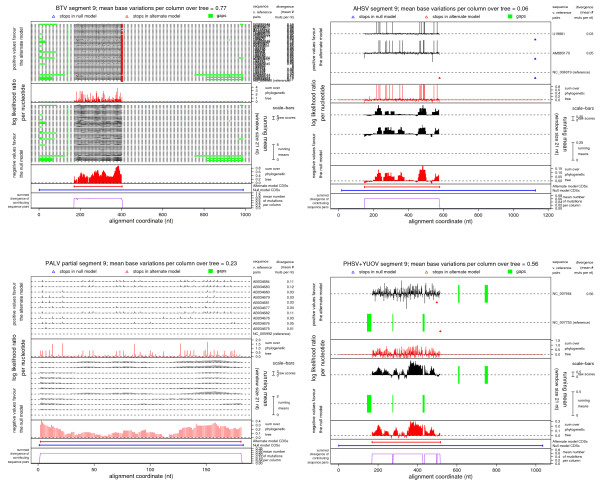
**MLOGD statistics for BTV, AHSV, PALV and PHSV/YUOV alignments**. Output plots from MLOGD used in the 'Test Query CDS' mode, applied to the ORFX region in BTV, AHSV, PALV and PHSV/YUOV sequence alignments. See [16] for full details of the MLOGD software. The null model comprises the VP6 CDS and the query CDS is ORFX. In each plot, the top panel displays the raw log(LR) statistics at each alignment position. There is a separate track for each reference – non-reference sequence pair (labelled at the right, together with the pairwise divergences; albeit not legible for the BTV alignment since it contains so many – i.e. 48 – sequences). Stop codons (of which there are none except 3' terminal ones) in each of the VP6 and ORFX reading frames, and alignment gaps for each sequence, are marked on the appropriate tracks. The second panel displays the Σ_tree _log(LR) statistic at each alignment position, where 'tree' represents a phylogenetic tree – see [16]. The third and fourth panels display sliding window means of the statistics in the first and second panels, respectively. The fifth panel shows the locations of the null and alternative model CDSs (i.e. VP6 and ORFX, respectively). The sixth panel shows the summed mean sequence divergence (base variations per alignment nt column) for the sequence pairs that contribute to the Σ_tree _log(LR) statistic at each alignment position. This is a measure of the information available at each alignment position (e.g. partially gapped regions have lower summed mean sequence divergence). The predominantly positive values in the fourth panel indicate that ORFX is subject to functional constraints, at the amino acid level, over the majority of its length.

The putative new CDS, ORFX, was first identified in a BTV sequence alignment, using MLOGD. In the RefSeq [GenBank: NC_006008] (1049 nt), ORFX has coords 182..415 (77 codons) and therefore is completely contained within the VP6 cistron (16..1005), overlapping it in the +1 reading frame (Figure 1). When applied to an alignment of 48 BTV sequences (see Methods; pairwise divergences ≤0.21 base variations per nucleotide and total alignment divergence ~0.77 independent base variations per column in the ORFX region), MLOGD detected a strong coding signature for ORFX (Figures 2, 3). There are ~180 independent base variations across the alignment in the ORFX region, thus providing MLOGD with a robust signal. Formally, and within the MLOGD model, *p *< 10^-40^. Indeed Figure 2 shows four non-overlapping – and hence completely independent – positively scoring windows in the ORFX region. Moreover, the MLOGD results showed that, within the ORFX region, ORFX is *more *conserved at the amino acid level than VP6 (Figure 2). Finally, inspection of the MLOGD output showed that the ORF is present in all of the 48 sequences (i.e. no premature termination codons; Figure 2).

### Nucleotide sequence analysis in BTV

In the 48-sequence BTV alignment (not shown), one can observe the following:

• The ORFX AUG initiation codon is present in all 48 sequences and is at the same location in the alignment. All have 'G' at +4; 46/48 have 'A' at -3 and 2/48 have 'G' at -3, giving the ORFX AUG codon a strong Kozak context [18].

• As far as amino acid constraints in the VP6 reading frame are concerned, there is no reason for the ORFX AUG codon to be conserved. In every sequence, the overlapping VP6-frame codons are gAU_Ggu. GAU codes for *Asp*, but *Asp *could also be encoded by GAC.

• Many sequences contain ORFX-frame termination codons just two codons 5' of the AUG codon. Thus initiation of ORFX at an upstream non-AUG codon, or via other non-canonical mechanisms, appears unlikely.

• ORFX is always in the +1 frame relative to the VP6 reading frame.

• The length of ORFX is 77 aa in 44/48 sequences (UAG termination codon) and 79 aa in 4/48 sequences (UAA termination codon). The alignment is gap-free within ORFX.

• All AUG codons upstream of the ORFX AUG codon are in the VP6 reading frame. There are a maximum of two upstream AUG codons in any given sequence, and the Kozak contexts of the upstream AUG codons are nearly always weak or medium (Table 1).

**Table 1:** Kozak contexts of VP6 AUG codons in BTV. Kozak contexts of AUG codons upstream of ORFX in BTV for the 34 segment 9 sequences which appear to contain the complete 5'UTR. Kozak contexts are assumed to be 'strong' if there is 'G' at +4 and an 'A' or 'G' at -3, 'medium' if one of these is present, and 'weak' if neither are present.

One upstream AUG codon				Two upstream AUG codons					
	
First		Strength	Number	First		Second		Strength	Number
-3	+4			-3	+4	-3	+4		
	
G	C	medium	5	C	U	U	G	weak-medium	15
A	A	medium	1	C	U	G	C	weak-medium	9
C	U	weak	1	C	U	A	A	weak-medium	1
				C	U	A	G	weak-strong	1
				C	U	C	C	weak-weak	1

**Figure 4:**
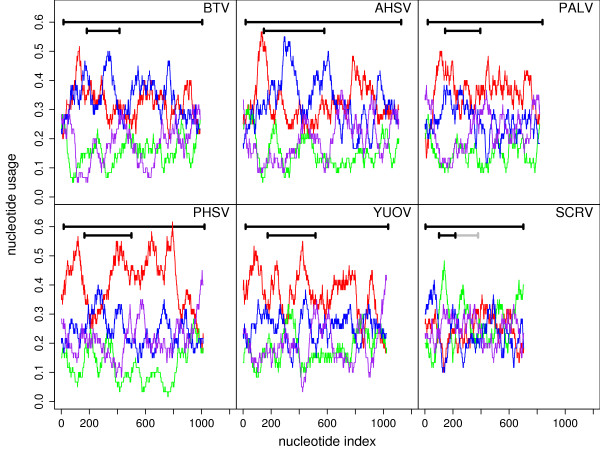
**Nucleotide frequencies for segment 9**. Nucleotide frequencies in 60 nt running windows along each *Orbivirus *segment 9 RefSeq. 'A' – red, 'C' – green, 'G' – blue, 'U' – purple. Horizontal black bars represent the locations of the VP6 CDS and ORFX (the grey bar represents ORFXb in SCRV). Except for SCRV, the sequences are A- or AG-rich, but they also have an A-rich peak just upstream of ORFX.

• There is only a single AUG codon (in a single sequence) in the purine-rich ~70 nt region (Figure 4) directly upstream of the ORFX AUG codon.

### Nucleotide sequence analysis in other Orbivirus RefSeqs

**Figure 5:**
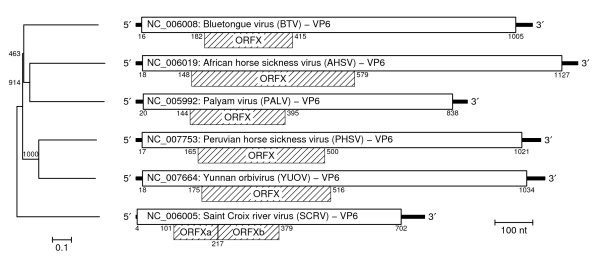
**Segment 9 genome maps for six Orbivirus species**. Genome maps for segment 9 of the six *Orbivirus *RefSeqs in GenBank, showing the location of putative ORFX homologues. In SCRV, no long ORF was found in the right location and frame; the two ORFs indicated here are separated by a stop codon. A phylogenetic tree for the six *Orbivirus *VP6 amino acid sequences (columns with alignment gaps excluded; neighbour-joining tree; numbers indicate bootstrap support [out of 1000]; scale bar represents the number of substitutions per site; tree produced with CLUSTALX [39]) is given at left.

The five non-BTV *Orbivirus *GenBank RefSeqs (see Methods) were inspected for a long ORF in the same location and reading frame as ORFX relative to the annotated VP6 CDS. Such an ORF was found in all RefSeqs except SCRV (Figure 5). The ORFX lengths are 143, 111, 113 and 83 codons in, respectively, AHSV, PHSV, YUOV and PALV. We propose (see Discussion) that ORFX is not present in SCRV. The following AUG codons are (potentially) used in the various RefSeqs (Kozak contexts – in parantheses – are assumed to be 'strong' if there is 'G' at +4 and an 'A' or 'G' at -3, 'medium' if one of these is present, and 'weak' if neither are present):

BTV: AUG1 (weak) and AUG2 (medium) in VP6 frame. AUG3 (strong) in ORFX frame. AUG[4-10] also in ORFX frame.

AHSV: AUG1 (weak) in VP6 frame. AUG2 (strong) in ORFX frame. AUG[3-10] also in ORFX frame.

PALV: AUG1 (weak) in VP6 frame. AUG2 (strong) in ORFX frame. AUG[3-7] also in ORFX frame.

PHSV: AUG1 (weak) in VP6 frame. AUG2 (medium) in ORFX frame (1 codon ORF). AUG3 (medium) in +2 frame (10 codon ORF). AUG4 (weak) and AUG5 (strong) in ORFX frame. AUG[6-7] also in ORFX frame.

YUOV: AUG1 (weak) in VP6 frame. AUG2 (medium) in ORFX frame (1 codon ORF). AUG3 (medium) in +2 frame (21 codon ORF; overlaps AUG4 [strong; +2 frame] and AUG5 [medium; VP6 frame]). AUG6 (medium), AUG7 (strong), AUG8 (strong) and AUG9 (medium) in ORFX frame.

SCRV: AUG1 (medium) and AUG2 (strong) in VP6 frame. AUG3 (medium) in ORFX frame (1 codon ORF). AUG4 (medium), AUG5 (strong) and AUG6 (strong) in VP6 frame. AUG7 (weak) and AUG8 (strong) in ORFX frame (ORFXa; Figure 5). AUG9 (weak) and AUG10 (weak) in ORFX frame (ORFXb; Figure 5).

### MLOGD analysis of ORFX coding potential

MLOGD can not be used effiectively on an alignment of the six RefSeqs because the pairwise divergences are too great. However it can be used on other within-species alignments. Alignments were constructed for (a) the 48 BTV sequences, (b) the 3 AHSV sequences, (c) the 11 PALV sequences (183 nt, partial), and (d) the PHSV and YUOV RefSeqs (see Methods). PHSV and YUOV are the two most-closely related of the six RefSeqs and are not too divergent for MLOGD. MLOGD results for ORFX are given in Table 2 and Figure 3. ORFX is present in all the aligned sequences (no premature termination codons) and, in each alignment, MLOGD detects a strong coding signature for ORFX. ORFX is longest in the three AHSV sequences – the maximal lengths being 143 codons in [Genbank:NC_006019], 154 codons in [Genbank:AM883170], and 169 codons in [Genbank:U19881].

**Table 2:** ORFX MLOGD statistics. MLOGD statistics for ORFX in different *Orbivirus *alignments. These statistics were derived using MLOGD in the 'Test Query CDS' mode (Figure 3) – specifically testing the coding potential of the whole ORFX – rather than the 'Sliding Window' mode used for Figure 2.

Species	Reference^1^	N_seqs_	Length	ln(LR)^2^	var/nt^3^	ln(LR)/nt^4^	${\text{N}}_{\mathrm{var}}^{5}$	${\text{div}}_{\max}^{6}$
BTV	NC_006008	48	234 nt	101.8	0.77	0.44	180	0.21
AHSV	NC_006019	3	429 nt	15.8	0.06	0.04	26	0.05
PALV	NC_005992	11	180^7 ^nt	29.7	0.23	0.16	41	0.12
PHSV/YUOV	NC_007753	2	336 nt	33.0	0.56	0.10	189	0.56

1. GenBank reference sequence used for MLOGD.

2. Total MLOGD log likelihood score – positive values indicate that ORFX is likely to be coding. Formally, exp(ln(LR)) gives $\frac{\text{P}(\text{alignment}|\text{ORFX coding})}{\text{P}(\text{alignment}|\text{ORFX noncoding})}$, which may be equated to $\frac{\text{P}(\text{ORFX coding})}{\text{P}(\text{ORFX noncoding})}$ if equal Bayesian priors are assumed. These probabilities are, however, subject to the assumptions of the MLOGD sequence evolution model [15]. Nonetheless, extensive tests with known single-coding and double-coding sequences indicate that 'N_var _≥ 20' and 'ln(LR)/nt ≥ $\frac{1}{6}$ × var/nt' signals robust detection of an overlapping same-strand CDS [16] (and unpublished data).

3. Alignment divergence per nucleotide – i.e. mean number of independent base variations per alignment column in the ORFX region.

4. Log likelihood score per alignment column.

5. Approximate total number of independent base variations in ORFX region.

6. Maximum pairwise divergence from the chosen reference sequence.

7. Alignment of PALV partial sequences – does not cover the entire ORFX region.

### Analysis of the ORFX peptide sequence

Application of blastp [19] to the ORFX peptide sequences for the six RefSeqs revealed no similar amino acid sequences in GenBank (14 Mar 2008), while tblastn identified only the ORFX region in other *Orbivirus *sequences (as expected). Application of InterProScan [20] to the six sequences returned no hits (protein motifs, domains etc).

The ORFX amino acid sequence appears to have greater amino acid conservation than the overlapping region of the VP6 CDS (e.g. Figure 2). In a comparison between [Genbank:NC_006008] and three divergent BTV sequences – [Genbank:DQ289044], [Genbank:D10905] and [Genbank:DQ825671], all three showed greater amino acid conservation (relative to NC_006008) in the ORFX frame than in the VP6 frame in the ORFX region. Specifically, there was respectively 87%, 78% and 100% amino acid identity in the ORFX frame, but only 58%, 73% and 83% identity in the VP6 frame. Similarly, in a comparison of [Genbank:NC_007753] (PHSV) with [Genbank:NC_007664] (YUOV), there were 32 amino acid identities in ORFX while, in the corresponding region of VP6, there were only 22 amino acid identities.

## Discussion

Due to the segmented nature of their genomes, the *Reoviridae *may escape a fundamental problem that many other eukaryotic viruses face – how to circumvent the host cell's general rule of 'one functional protein per mRNA'. Nonetheless, of the 352 *Reoviridae *RefSeqs in GenBank (10 Mar 2008; 33 species × 9–12 segments per species), ~5% are multicistronic. Among these are a few examples of fully overlapping genes apparently translated via leaky scanning, for example in *Phytoreovirus *segment S12 or S9 [21] and mammalian *Orthoreovirus *segment S1 [22,23].

For optimal leaky scanning [24], one would expect the VP6 CDS to initiate at AUG1 with weak context and ORFX to initiate at AUG2 with strong context. This indeed is the situation in the AHSV and PALV RefSeqs. Although there are two upstream VP6-frame AUG codons in many BTV serotypes, leaky scanning still appears fairly straightforward in this virus as a translational mechanism for ORFX (though potentially at a much lower abundance than VP6). In the YUOV and PHSV RefSeqs, leaky scanning may be possible, but requires scanning through or translation and reinitiation of two upstream short ORFs. It is interesting, and possibly relevant, that in another *Reoviridae *species – Avian reovirus – a novel, as yet not fully understood, scanning-independent ribosome migration mechanism is used to bypass two upstream CDSs in order to translate the 3'-proximal CDS on the tricistronic S1 mRNA [25,26].

IRESs have not been reported in the *Reoviridae *and, at this genomic location, use of an IRES would seem unlikely. However, it has been shown that a variety of poly-purine A-rich sequences – such as (GAAA)_16 _– can serve as efficient IRESs without the requirement for a complex RNA secondary structure such as in the *Picornaviridae *IRESs [27], so it is interesting to note that there is an A-rich poly-purine tract just upstream of ORFX in all species except SCRV (Figure 4). In the BTV RefSeq, for example, the 68 nt immediately preceding ORFX comprise 32 A, 7 C, 25 G and 4 U nucleotides. In fact the entire sequences (except SCRV) are A- or AG-rich (Table 3). Nonetheless the region just upstream of ORFX is a peak in A-richness (Figure 4). Admittedly, this could be due to many other reasons (e.g. just amino acid coding constraints in VP6) and there is no strong reason to suspect an IRES here.

**Table 3:** Nucleotide frequencies for segment 9. Mean nucleotide frequencies for the six *Orbivirus *segment 9 RefSeqs in GenBank.

RefSeq	Species	A%	C%	G%	U%
NC_006008	BTV	32	16	33	19
NC_006019	AHSV	32	16	32	20
NC_005992	PALV	36	16	26	23
NC_007753	PHSV	41	13	24	22
NC_007664	YUOV	36	18	25	20
NC_006005	SCRV	25	27	24	25

SCRV lacks a long ORF in the correct reading frame and location for an ORFX homologue. The number (six) and contexts (3 are strong) of upstream AUG codons make conventional leaky scanning to 'ORFXa' (38 codons; Figure 5) extremely unlikely. It is quite possible, therefore, that no ORFX homologue is present in SCRV. This is not too surprising – SCRV segment 9 is the most divergent, and the shortest, of the six RefSeqs (Figure 5) [28]. SCRV is also the only species of the six which is tick-borne instead of insect-borne (BTV, AHSV and PALV are transmitted by midges; YUOV by mosquitoes).

At ~9.5 kDa, the putative ORFX product in BTV is too small to appear on most published protein gels. Nonetheless there are unidentified low molecular mass bands in a number of reported gels [29-32], often running near the dye front, that *may *represent ORFX product. Furthermore, ref. [33] (*in vitro *translation of the individual segments) noted, with reference to excluded data, that segment 9 may encode a low molecular weight protein in addition to VP6.

The ORFX product is largest in AHSV (~17 kDa in [GenBank:NC_006019] and ~20 kDa in [GenBank:U19881]). Ref. [34] (*in vitro *translation of the individual AHSV segments, and comparison with proteins extracted from infected cell lysate) clearly identified an additional non-structural protein translated from segment 9 – termed 'NS3' – migrating ~1.5 kDa behind the 'NS4/4A' proteins (equivalent to NS3/3A in our notation) translated from segment 10. 'NS3' is a good candidate for ORFX product migrating a little slower than expected, possibly as a result of post-translational modification. The protein labelled 'VP6' in ref. [34] appears to be a truncated version of VP5 (translated from the same segment as VP5, and both were shown to have similar partial protease digestion products). Interestingly the VP6 protein (our notation) is not visible as a product of segment 9 translation in Fig. 6 of ref. [34], but may be visible in Fig. 7 of ref. [34] (migrating next to NS2), unless this is cross-contamination. An additional segment 9 product (~20 kDa), migrating ahead of 'NS4/4A', is also visible (albeit fainter) in Fig. 7 of ref. [34]. If the 'NS3' band is post-translationally modified ORFX product, then this band could be unmodified ORFX product.

Ref. [35] also identified a number of low molecular mass proteins in AHSV-infected cells – in particular P23, P20 and P21. Ref. [35] equated two of these (P20 and P21) to the segment 10 products NS3/3A (~24/~22 kDa in AHSV). The third protein may be ORFX product.

In addition to its small size, the fact that ORFX product has not been widely reported suggests that it may be present only in low abundance and/or only expressed at certain stages (e.g. only in the insect vector) or cellular locations.

## Conclusion

We have identified a conserved ORF (ORFX) overlapping the *Orbivirus *VP6 CDS in the +1 reading frame. ORFX ranges from 77–169 codons in length, depending on species, and is present in all *Orbivirus *segment 9 sequences analysed except for the highly divergent species SCRV. The software package MLOGD – designed specifically for identifying and analysing overlapping CDSs – finds a strong coding signature for ORFX when applied to BTV, AHSV, PALV and PHSV/YUOV sequence alignments. The location and Kozak context of the VP6 and ORFX initiation codons is generally consistent with a leaky scanning model for ORFX translation. ORFX product bears no homology to known proteins.

We hope that presentation of this bioinformatic analysis will stimulate an attempt to experimentally verify the expression and functional role of ORFX product. Initial verification could be by means of immunoblotting with ORFX-specific antibodies or gel purification of ORFX product from virus-infected cell protein extracts, followed by mass spectrometry.

## Methods

In GenBank, there are whole-genome RefSeqs for six *Orbivirus *species: Bluetongue virus (BTV), African horse sickness virus (AHSV), Peruvian horse sickness virus (PHSV), Yunnan orbivirus (YUOV), Palyam virus (PALV) and Saint Croix river virus (SCRV). All six genomes comprise 10 segments. The segments homologous to BTV segment 9 (encoding VP6) were identified by finding the best blastp-match, among the 10 BTV translated segments, for the longest ORF in each of the 50 non-BTV segments. The identifications were verified, where possible, by information in the GenBank-file headers and in the literature (AHSV [36]; YUOV [37]; PALV [38]; SCRV [28]).

As of 11 May 2007, there were 1273 *Orbivirus *sequences in GenBank (i.e. including partial sequences), however most of these are not segment 9. Incidently, none of these sequences has more than one CDS annotated. Segment 9 sequences were extracted (a) using the GenBank-file DEFINITION headers, and (b) by finding the best blastp-match for the longest ORF in each sequence among the 10 BTV translated segments. These were supplemented with all GenBank (16 Mar 2008) tblastn matches to the ORFX peptide sequences from the six RefSeqs (providing one additional recent sequence). After removing duplicate sequences, the following segment 9 sequences were found: (1) the 6 RefSeqs for BTV, AHSV, PHSV, YUOV, PALV and SCRV (all complete); (2) 47 other BTV sequences (mostly complete VP6 CDS; all cover ORFX completely; ~34 contain the full 5' UTR); (3) 2 other AHSV sequences (full genome); and (4) 10 PALV partial sequences (183 nt, completely contained in the ORFX region).

The GenBank accession numbers are as follows: BTV – NC_006008, A22393, AF403418, AF403419, AF403420, AF403421, AF403423, AY124373, AY493691, D10905, DQ289041, DQ289042, DQ289043, DQ289044, DQ289045, DQ289046, DQ289047, DQ289048, DQ289050, DQ825668, DQ825669, DQ825671, DQ832170, L08668, L08669, L08670, L08671, L08672, U55778, U55779, U55780, U55781, U55782, U55784, U55785, U55786, U55787, U55788, U55790, U55792, U55793, U55794, U55795, U55796, U55797, U55799, U55800, U55801; AHSV – NC_006019, U19881, AM883170; PHSV – NC_007753; YUOV – NC_007664; PALV – NC_005992, AB034675, AB034676, AB034677, AB034678, AB034679, AB034680, AB034681, AB034682, AB034683, AB034684; SCRV – NC_006005.

## Competing interests

The author(s) declare that they have no competing interests.

## Authors' contributions

AEF carried out the bioinformatics analyses and wrote the manuscript.
